# Suppression of pullulanase-induced cytotoxic T cell response with a dual promoter in GSD IIIa mice

**DOI:** 10.1172/jci.insight.152970

**Published:** 2022-12-08

**Authors:** Jeong-A Lim, Priya S. Kishnani, Baodong Sun

**Affiliations:** Division of Medical Genetics, Department of Pediatrics, Duke University School of Medicine, Durham, North Carolina, USA.

**Keywords:** Genetics, Therapeutics, Gene therapy, Genetic diseases, Glucose metabolism

## Abstract

Glycogen debranching enzyme deficiency in glycogen storage disease type III (GSD III) results in excessive glycogen accumulation in multiple tissues, primarily the liver, heart, and skeletal muscle. We recently reported that an adeno-associated virus vector expressing a bacterial debranching enzyme (pullulanase) driven by the ubiquitous CMV enhancer/chicken β-actin (CB) promoter cleared glycogen in major affected tissues of infant GSD IIIa mice. In this study, we developed a potentially novel dual promoter consisting of a liver-specific promoter (LSP) and the CB promoter for gene therapy in adult GSD IIIa mice. Ten-week treatment with an adeno-associated virus vector containing the LSP-CB dual promoter in adult GSD IIIa mice significantly increased pullulanase expression and reduced glycogen contents in the liver, heart, and skeletal muscle, accompanied by the reversal of liver fibrosis, improved muscle function, and a significant decrease in plasma biomarkers alanine aminotransferase, aspartate aminotransferase, and creatine kinase. Compared with the CB promoter, the dual promoter effectively decreased pullulanase-induced cytotoxic T lymphocyte responses and enabled persistent therapeutic gene expression in adult GSD IIIa mice. Future studies are needed to determine the long-term durability of dual promoter–mediated expression of pullulanase in adult GSD IIIa mice and in large animal models.

## Graphical abstract

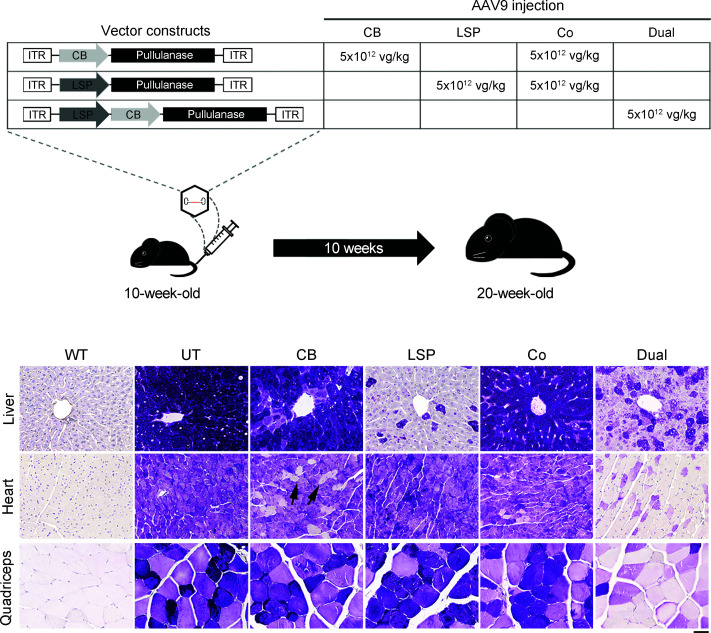

## Introduction

Glycogen storage disease type III (GSD III) is an autosomal recessive disorder caused by pathogenic variants in the *AGL* gene, which encodes the glycogen debranching enzyme (GDE), a key enzyme responsible for glycogen degradation. Human GDE has 2 independent catalytic activities, a 4-α-glucanotransferase (Enzyme Commission [EC] 2.4.1.25) activity and an amylo-1, 6-glucosidase (EC 3.2.1.33) activity, in the same polypeptide (1). Loss of GDE function leads to excessive accumulation of abnormal glycogen (limit dextrin) in multiple tissues, primarily the liver, heart, and skeletal muscles. Most individuals (~85%) have both muscle and liver involvement (GSD IIIa), whereas a small subset (~15%) have disease limited to the liver (GSD IIIb). Patients present within the first year of life with significant hepatomegaly, hypoglycemia, and hyperlipidemia as well as elevated hepatic transaminases and elevated creatine kinase (CK). Long-term complications include progressive liver fibrosis with a risk for hepatic failure, and some patients develop end-stage liver cirrhosis or hepatocellular carcinomas (2–5). Progressive myopathy and cardiomyopathy are the major causes of morbidity and mortality. Muscle weakness and myopathy begin in the first decade of life and become more prominent as patients get older; some patients can become wheelchair dependent due to severe skeletal muscle function impairment. Ventricular hypertrophy is a frequent finding, and sudden deaths due to life-threatening cardiac arrhythmias or cardiac failure have been reported (6–10).

To date, no curative treatment is available for GSD III, and current symptomatic dietary management does not prevent ongoing disease progression. Liver transplantation is indicated for patients with severe hepatic cirrhosis and/or hepatocellular carcinoma; progressive myopathy persists and represents a significant, unmet need.

Because GSD III is a single-gene disorder, gene therapy with adeno-associated virus (AAV) vectors provides an optimal treatment approach, as AAV, especially AAV serotype 9 (AAV9), can reliably transduce both liver and muscle tissues with high efficiency (11, 12). In the past decade, AAV has become the most commonly used gene delivery vector in clinical trials for a broad range of human genetic diseases, especially disorders of inborn errors of metabolism (13). A major challenge for the development of AAV-mediated gene therapy for GSD III is the inability of an AAV vector to carry the large-sized (4.6 kb) human *AGL* cDNA, due to its small packaging capacity. To overcome this limitation, we recently reported a gene therapy method using an AAV vector expressing a smaller bacterial GDE, pullulanase (type I, EC 3.2.1.41) derived from Bacillus subtilis strain 168, in infant GSD IIIa mice (14). Unlike human GDE, which has both 4-α-glucanotransferase activity and amylo-1,6-glucosidase activity, pullulanase has only amylo-α-1, 6-glucosidase activity and can directly cleave the α(1*→*6) bonds at the branching points in limit dextrin, thus allowing glycogen phosphorylase to continually break down glycogen molecules (15, 16). Intravenous injection of an AAV9 vector containing a 2.2 kb codon-optimized pullulanase cDNA driven by the ubiquitous CMV enhancer/chicken β-actin (CB) promoter (AAV-CB-Pull) into 2-week-old infant GSD IIIa mice significantly decreased glycogen accumulation in the heart and skeletal muscles but not in the liver and significantly improved muscle function after 10 weeks (14). However, following intravenous injection of the AAV-CB-Pull vector in adult GSD IIIa mice, pullulanase expression was detected in the liver, heart, and skeletal muscle after 2 weeks and completely disappeared after 7 weeks (our unpublished observations), likely due to transgene-induced cytotoxic T lymphocyte (CTL) response. This suggests that pullulanase-induced immune responses must be addressed in animal models in order to translate this gene therapy approach to patients.

It is known that using tissue-specific promoters is an effective approach for evading transgene-induced immune responses to allow sustained therapeutic transgene expression. The induction of immunotolerance to the transgene products by AAV vectors containing a liver-specific promoter (LSP) has been well documented in hemophilia preclinical studies and clinical trials (17–21). We have developed a liver-restricted gene therapy approach for Pompe disease using an AAV8 vector containing a LSP to express human acid α-glucosidase (hGAA), which can suppress both humoral and cellular immune responses to hGAA product and correct GAA deficiency in the muscle and brain through mannose-6-phosphate receptor–mediated uptake of secreted hGAA precursor from the blood in GAA-KO mice (22, 23). This gene therapy approach is currently being investigated in a phase I clinical trial (NCT03533673) involving patients with late-onset Pompe disease (24, 25). We also demonstrated that muscle-restricted gene therapy with AAV vectors containing muscle-specific promoters prevented CTL response to hGAA and cleared glycogen storage in the hearts and skeletal muscles of GAA-KO mice (26, 27). However, these approaches with a tissue-specific promoter express the transgene in one tissue, which is not ideal for treatment of GSD III that involves multiple tissues.

Although muscle and liver are the major affected tissues, glycogen accumulation is also observed in other organs, including the CNS in GSD IIIa mice (14). Thus, an AAV vector featuring reduced immunogenicity, while allowing pullulanase expression in all affected tissues, is desired for treatment of GSD III. In this study, we developed a LSP-CB dual promoter and tested its ability to suppress CTL response to pullulanase and to enable sustained expression of the therapeutic enzyme in major affected tissues of GSD IIIa mice.

## Results

**Figure 1:**
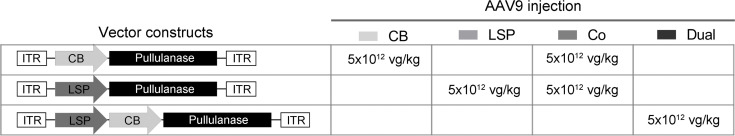
Experimental design and pullulanase expression by the LSP-CB dual promoter in major affected tissues of GSD IIIa mice. The diagram shows the injection doses and the constructs of AAV vectors containing a 2.2 kb codon-optimized pullulanase cDNA under the control of the ubiquitous CMV enhancer/chicken β-actin (CB) promoter, the liver-specific promoter (LSP), and the LSP-CB dual promoter. For AAV injection, CB, mice injected with AAV9-CB-Pull vector; LSP, mice injected with AAV9-LSP-Pull; Co, mice coinjected with AAV9-CB-Pull and AAV9-LSP-Pull; Dual, mice injected with AAV9-Dual-Pull. The AAV vectors were packaged into AAV9 and intravenously injected into 10-week-old GSD IIIa mice at the same dose of 5 × 10^12^ vg/kg. Mice were euthanized after 2 or 10 weeks.

Three AAV constructs carrying the codon-optimized pullulanase cDNA under the control of the ubiquitous CB promoter (AAV-CB-Pull), a LSP (AAV-LSP-Pull), or a LSP-CB dual promoter (AAV-Dual-Pull) were packaged into AAV9 (Figure 1). Ten-week-old GSD IIIa mice were systemically injected with each of the AAV vectors (CB, LSP, and dual) or coinjected with the AAV-LSP-Pull and AAV-CB-Pull vectors at the same dose of 5 × 10^12^ vector genomes per kilogram (vg/kg) (Figure 1). Tissues, including the liver, heart, skeletal muscles, smooth muscles, brain, and spinal cord, were harvested after 2 or 10 weeks.

### All 4 AAV treatments cleared glycogen accumulation in liver but only AAV-Dual-Pull also significantly decreased glycogen levels in heart and skeletal muscle after 2 weeks of treatment.

**Figure 2:**
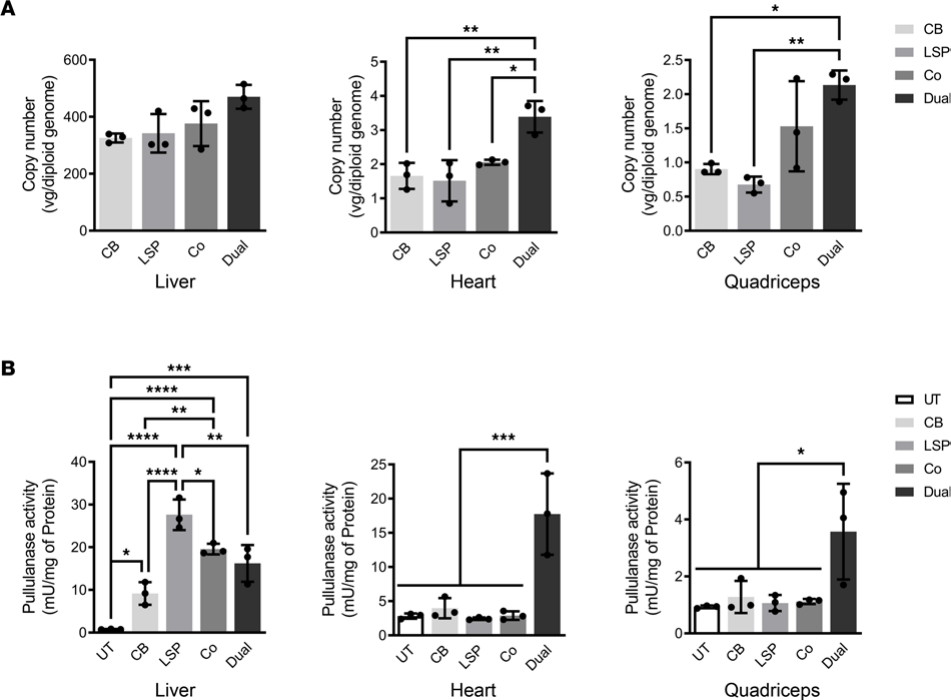
AAV biodistribution and pullulanase expression in major affected tissues after 2 weeks of AAV treatment. (**A**) AAV genome copy numbers were determined by real-time PCR in the liver, heart, and quadriceps. Data are shown as the mean ± SD; *n* = 3 for each group. Kruskal-Wallis test for liver; ordinary 1-way ANOVA for heart and quadriceps. *P < 0.05, **P < 0.01. (**B**) Pullulanase activity was assayed in the liver, heart, and skeletal muscle. Data are shown as the mean ± SD; *n* = 3 for each group. Ordinary 1-way ANOVA; *P < 0.05, **P < 0.01, ***P < 0.001, ****P < 0.0001.

**Figure 3:**
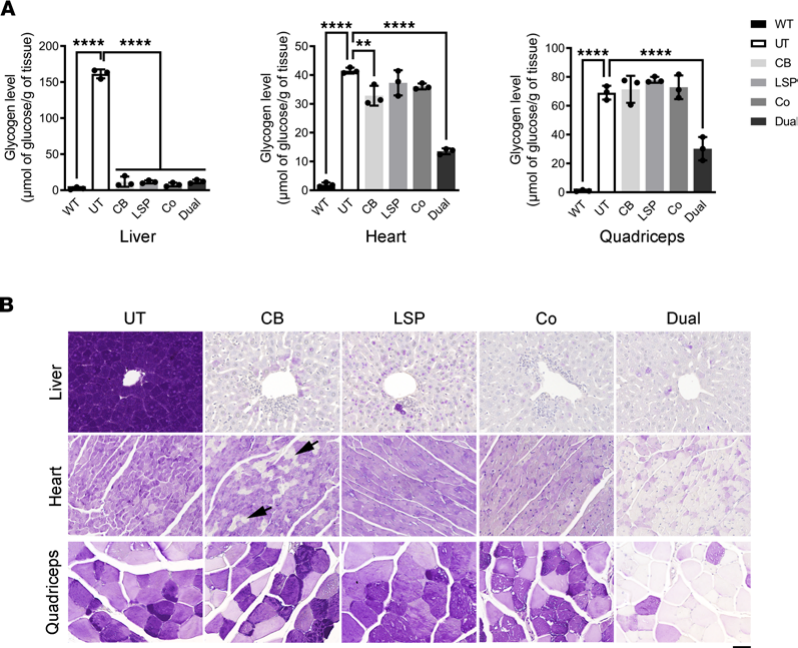
Glycogen clearance in major affected tissues after 2 weeks of AAV treatment. (**A**) Glycogen contents were measured in the liver, heart, and skeletal muscle. Data are shown as the mean ± SD; *n* = 3 for each group. Ordinary 1-way ANOVA; **P < 0.01, ****P < 0.0001 versus UT. (**B**) Periodic acid–Schiff staining of tissue sections was performed to confirm the glycogen content results. Glycogen was stained in purple. The arrows pointed out the glycogen-free cardiac cells occasionally seen in the CB-treated heart. Images are representative of at least 3 mice in each group. Scale bar: 50 μm. UT, untreated.

At 2 weeks after vector injection, extremely high copy numbers of AAV genomes were detected in the livers of all 4 treatment groups (Figure 2A). In heart and quadriceps, all 4 AAV treatments resulted in detectable AAV genomes, but higher AAV genome numbers were observed in the AAV-Dual-Pull–treated mice (Figure 2A). All 4 AAV treatments significantly increased pullulanase activities in the liver, but only the AAV-Dual-Pull treatment significantly increased pullulanase activities in the heart and quadriceps, compared with the untreated (UT) and other AAV-treated mice (Figure 2B). Consistent with the pullulanase activity results, all 4 AAV treatments dramatically reduced liver glycogen accumulation to near WT levels (Figure 3A), a finding that was further confirmed by Periodic acid–Schiff (PAS) staining of liver sections, which showed that massive purple-stained glycogen observed in the UT liver was cleared in all the AAV-treated livers (Figure 3B). Neither the AAV-LSP-Pull nor the cotreatment alone significantly reduced glycogen accumulation in the heart and skeletal muscle, compared with UT heart and skeletal muscle (Figure 3). The AAV-CB-Pull–treated heart demonstrated a minor (~20%) reduction in glycogen content in the heart (Figure 3A), accompanied by the clearance of glycogen accumulation in some of the cardiomyocytes (Figure 3B, black arrows). Notably, only the AAV-Dual-Pull treatment significantly reduced glycogen levels and markedly cleared glycogen accumulation in the heart and skeletal muscle (Figure 3).

### LSP-CB dual promoter resulted in persistent pullulanase expression and glycogen reduction in all major affected tissues after 10 weeks of AAV treatment.

**Figure 4:**
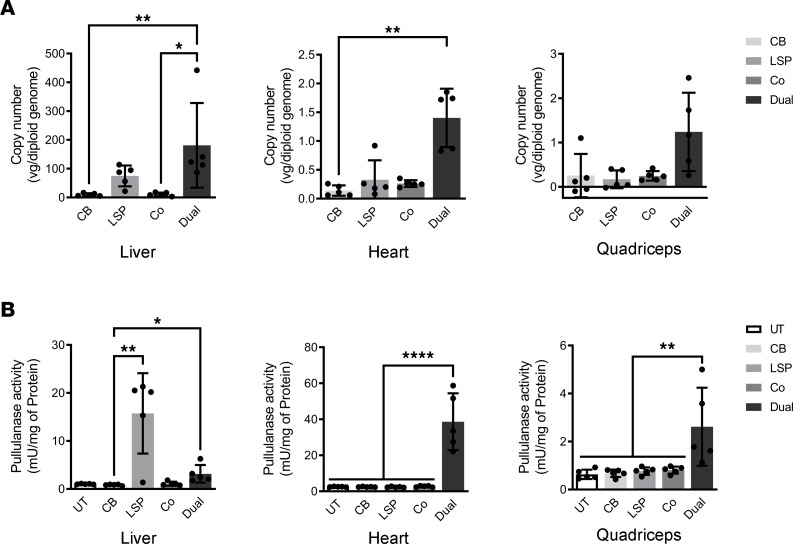
LSP-CB dual promoter enabled sustained pullulanase expression in major affected tissues after 10 weeks of AAV treatment. (**A**) AAV genome copy numbers were determined by real-time PCR using gene-specific primers for pullulanase in the liver, heart, and skeletal muscle (quadriceps) after 10 weeks of treatment. Data are shown as the mean ± SD; *n* = 5 for each group. Kruskal-Wallis tests; *P < 0.05, **P < 0.01. (**B**) Pullulanase activities were evaluated in the liver, heart, and quadriceps after 10 weeks of treatment. Data are shown as the mean ± SD; *n* = 5 for each group. Kruskal-Wallis test for liver; ordinary 1-way ANOVA for heart and quadriceps; *P < 0.05, **P < 0.01, ****P < 0.0001.

At 10 weeks after vector injection, AAV vg were remarkably decreased in the liver, heart, and quadriceps treated with AAV-CB-Pull or with coadministration of AAV-LSP-Pull and AAV-CB-Pull but remained high in the AAV-Dual-Pull–treated tissues. The AAV-LSP-Pull–treated mice showed relatively high vg in the liver but not in the heart and skeletal muscle (Figure 4A). As shown in Figure 4B, pullulanase activities were significantly elevated in the heart and quadriceps and moderately increased in the liver of the AAV-Dual-Pull–treated mice. In contrast, pullulanase activity was significantly increased in only the liver of AAV-LSP-Pull–treated mice. Pullulanase activity was not significantly elevated in any tested tissues of the AAV-CB-Pull–treated or cotreated mice. The AAV-LSP-Pull–treated liver showed lower vg but higher pullulanase activity than the AAV-Dual-Pull–treated liver, suggesting that the LSP promoter is more active than the dual promoter in liver (Figure 4).

**Figure 5:**
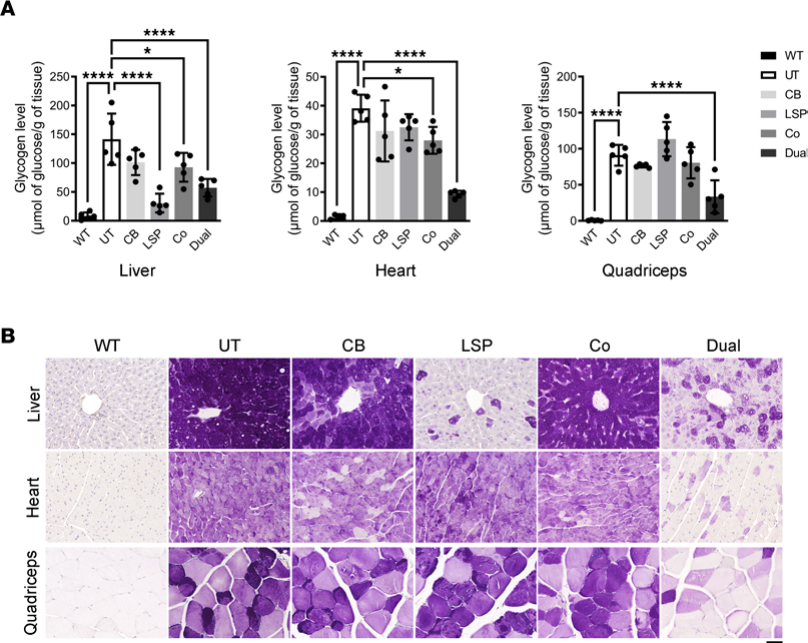
AAV-Dual-Pull reduced glycogen accumulation in the liver, heart, and skeletal muscle after 10 weeks of treatment. (**A**) Glycogen contents were measured in the liver, heart, and skeletal muscle. WT, age-matched WT mice. Data are shown as the mean ± SD; *n* = 5 for each group. Ordinary 1-way ANOVA; *P < 0.05, ****P < 0.0001 versus UT. (**B**) PAS staining of tissue sections was performed to confirm the results of glycogen content assay. No glycogen accumulation was observed in any tissues of the WT mice. Images are representative of at least 3 mice in each group. Scale bar: 50 μm. UT, untreated.

After 10 weeks of treatment, liver glycogen content was slightly increased in the AAV-LSP-Pull–treated mice and moderately increased in the AAV-Dual-Pull–treated mice from the 2-week treatment time point, but it still remained significantly reduced (–78% by AAV-LSP-Pull and –60% by AAV-Dual-Pull) compared with UT mice. In contrast, liver glycogen content returned to the near UT level in the AAV-CB-Pull–treated mice (*P* > 0.05) (Figure 5A). The cotreatment significantly reduced glycogen content in the liver (–34%) and heart (–28%) but not in the quadriceps compared with that in UT mice. Neither AAV-CB-Pull nor AAV-LSP-Pull significantly reduced glycogen content in the heart or skeletal muscle, but the AAV-Dual-Pull treatment significantly decreased glycogen content in both tissues (–76% in heart and –63% in quadriceps) (Figure 5A). The glycogen content results were further confirmed by PAS staining of tissues (Figure 5B). Further analysis of other tissues by PAS staining showed that the AAV-Dual-Pull treatment cleared glycogen storage in other skeletal muscles (diaphragm and soleus muscle) and the kidney, but it had no effect on the smooth muscles (bladder and small intestine) and the CNS (brain and spinal cord) (Supplemental Figure 1; supplemental material available online with this article; https://doi.org/10.1172/jci.insight.152970DS1).

### Ten-week treatment with AAV-Dual-Pull–ameliorated liver abnormalities, reversed hepatic fibrosis, and improved muscle function.

**Figure 6:**
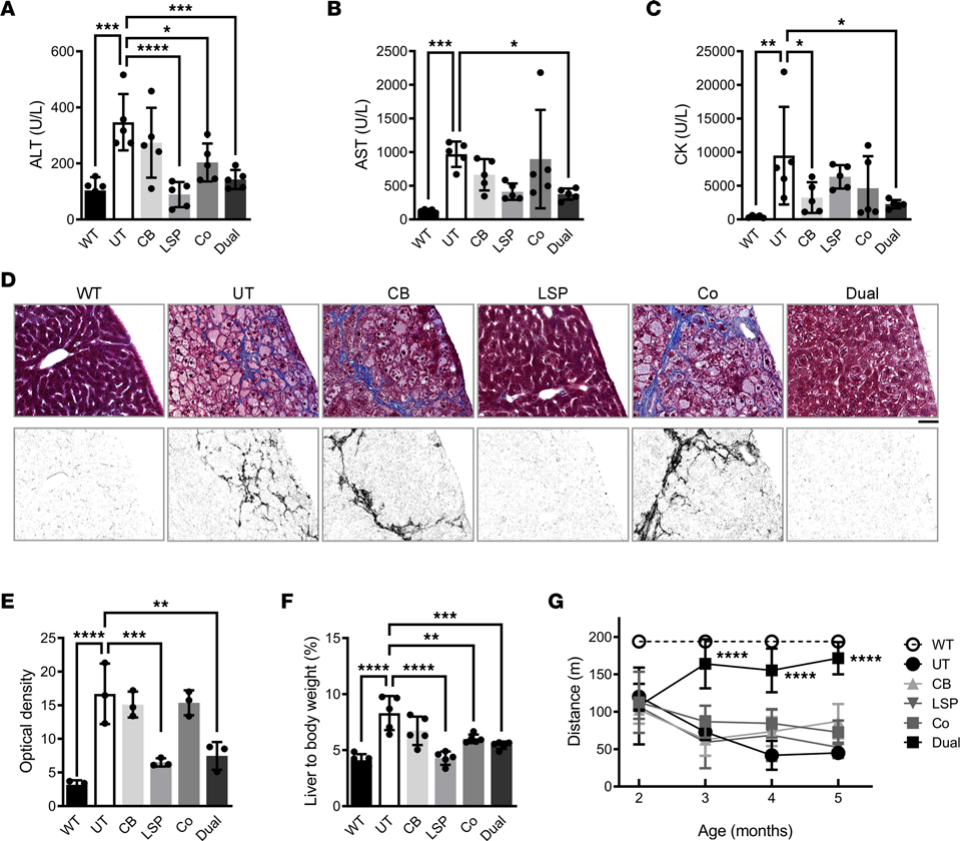
AAV-Dual-Pull recovered liver abnormalities and improved muscle function after 10 weeks of treatment. (**A–C**) Plasma alanine aminotransferase (ALT), aspartate aminotransferase (AST), and creatine kinase (CK) activities were measured to evaluate liver and muscle damage. Data are shown as the mean ± SD; *n* = 5 for each group. Ordinary 1-way ANOVA for ALT (**A**) and CK (**C**); Kruskal-Wallis test for AST (**B**); *P < 0.05, **P < 0.01, ***P < 0.001, ****P < 0.0001 versus UT. (**D**) Trichrome staining of liver sections was performed for the detection of liver fibrosis. Substantial fibrotic tissues (blue) were observed in the livers of UT, chicken β-actin–treated (CB-treated), and cotreated mice (top). Separated blue-stained areas from the trichrome images are shown in black and white by ImageJ using the threshold color option. At least 3 mice in each group were examined, and representative images are shown (bottom). Scale bar: 50 μm. (**E**) Quantitative analysis of the black-and-white images in **D** using ImageJ. The optical density of each image was measured for the quantitation of liver fibrosis. Three different areas of each mouse from *n* = 3 mice in each group were examined. Data are shown as the mean ± SD. Ordinary 1-way ANOVA; **P < 0.01, ***P < 0.001, ****P < 0.0001 versus UT. (**F**) The ratio of liver to body weight was measured to determine liver size (hepatomegaly). Data are shown as the mean ± SD; *n* = 5 for each group. Ordinary 1-way ANOVA; **P < 0.01, ***P < 0.001, ****P < 0.0001 versus UT. (**G**) Treadmill test was used to evaluate exercise intolerance for the UT and AAV-treated GSD IIIa mice. The graph represents the maximum running distance. Data are shown as the mean ± SD; *n* = 5 for each group. Ordinary 1-way ANOVA, ****P < 0.0001 versus UT. UT, untreated.

Ten weeks after AAV injection, AAV-LSP-Pull, AAV-Dual-Pull, and cotreatment significantly decreased plasma activities of liver transaminase alanine aminotransferase (ALT) compared with those in UT mice (Figure 6A), but only the AAV-Dual-Pull treatment also significantly decreased the aspartate aminotransferase (AST) (Figure 6B) and CK activity (Figure 6C). Interestingly, the AAV-CB-Pull treatment significantly decreased CK activity but had no effect on ALT and AST (Figure 6, A–C). Liver fibrosis was evaluated by Masson’s trichrome staining of liver sections. Hepatic fibrosis can be seen in 10-week-old UT GSD IIIa mice (pretreatment, data not shown). After 10 weeks of gene therapy, many blue-stained fibrotic tissues were observed in the UT, AAV-CB-Pull–treated, or AAV-LSP-Pull and AAV-CB-Pull cotreated livers but were almost invisible in the WT, AAV-LSP-Pull–treated, and AAV-Dual-Pull–treated livers (Figure 6D). Semiquantitation of the blue-stained fibrotic tissues in the liver sections by ImageJ showed that the UT, AAV-CB-Pull–treated, or cotreated livers had similarly high levels of fibrosis, while the AAV-LSP-Pull and AAV-Dual-Pull–treated livers showed significantly lowered fibrosis (Figure 6E). Liver size was determined by measuring the ratio of liver to body weight. With the exception of AAV-CB-Pull, the other 3 treatments significantly decreased the liver size to the range observed for WT livers (Figure 6F). Muscle function was evaluated by the treadmill test during the course of 10-week AAV treatment. Only the AAV-Dual-Pull–treated mice demonstrated improvement in the running distance compared with the UT and other AAV-treated mice (Figure 6G).

### Both LSP and dual promoter effectively suppressed CTL responses against pullulanase in adult GSD IIIa mice.

**Figure 7:**
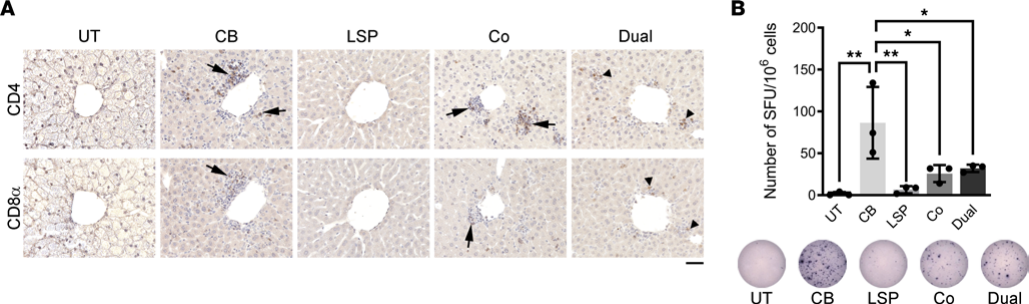
Both the LSP and LSP-CB dual promoter decreased pullulanase-induced CTL response in GSD IIIa mice. (**A**) Paraffin-embedded liver sections from GSD IIIa mice 2 weeks after AAV injection were stained with an anti-CD4^+^ or anti-CD8α antibody to detect cytotoxic T cell responses. Infiltrations of CD4^+^ or CD8α^+^ lymphocytes (brown stained, arrows) were abundant in the CB-treated livers, moderately present in the cotreated livers. CD4^+^ or CD8α^+^ cells (arrowheads) were occasionally seen in the dual-treated livers and almost absent in the LSP-treated livers. Images are representative of at least 3 mice in each group. Scale bar: 50 μm. (**B**) Mouse IFN-γ ELISpot was performed to detect cell-mediated immune responses against pullulanase. Splenocytes isolated 2 weeks after AAV injection were stimulated by the final pullulanase peptides pool (Supplemental Figure 2). The levels of pullulanase-induced IFN-γ secretion were determined by the number of spot-forming units (SFU) per million splenocytes and are represented in the graph. The number of SFU was the highest in the CB-treated mice, significantly decreased in the dual- and cotreated mice, and the lowest in the LSP-treated mice. Data are shown as the mean ± SD; *n* = 3, triplicates. Ordinary 1-way ANOVA; *P < 0.05, **P < 0.01.

To evaluate transgene-related T cell immune responses, we first performed immunohistochemical staining of liver sections with an anti-CD4^+^ or anti-CD8α antibody 2 weeks after AAV injection. As shown in Figure 7A, CD4^+^ and CD8^+^ lymphocyte infiltrations were abundant in the AAV-CB-Pull–treated liver, moderately present in the cotreated liver, occasionally seen in the AAV-Dual-Pull–treated liver, and almost absent in the AAV-LSP-Pull–treated liver. We next performed a mouse IFN-γ ELISpot assay using splenocytes from GSD IIIa mice 2 weeks after AAV treatment to analyze pullulanase-specific CTL responses. A peptide library covering the entire pullulanase sequence (total of 142 peptides) was designed to consist of 15-mer peptides, each overlapping by 10 amino acids with adjacent peptides. Twenty-four pullulanase peptide pools each containing 11–12 peptides (Supplemental Figure 2) were screened by 2 rounds of IFN-γ ELISpot assays using the CB-treated splenocytes (positive controls). A final peptide pool that contained 8 of the most immunogenic peptides was used to compare the pullulanase-specific T cell responses to the AAV treatments. The numbers of IFN-γ spot-forming units (SFU) per 10^6^ splenocytes were used to quantify the CTL responses against pullulanase. Consistent with the immunohistochemistry results, the CB-treated mice showed the highest numbers of SFU, and the LSP-treated mice had the lowest numbers of SFU (8% of that in CB-treated mice). Interestingly, the numbers of SFU from splenocytes were similarly decreased in the dual-treated mice (–61%) and cotreated mice (–69%) (Figure 7B), even though the immunohistochemical data suggested that the cotreatment caused more T cell responses than the dual treatment in the liver (Figure 7A). Taken together, these data suggested that both the LSP and the dual promoter effectively suppressed pullulanase-induced CTL responses in adult GSD IIIa mice.

## Discussion

Deficiency of GDE results in excessive accumulation of abnormally structured glycogen (limit dextrin) primarily in the liver, heart, and skeletal muscle in patients with GSD IIIa. In GSD IIIa mice, glycogen accumulation was observed at high levels in the liver, heart, skeletal muscles (quadriceps, gastrocnemius, soleus, and diaphragm), and smooth muscles (bladder and intestine) and moderately in the kidney and in some regions of the brain and spinal cord (14, 28). Currently, there is no definitive treatment for the disease. Enzyme replacement therapy with human GDE is not feasible due to the lack of a natural receptor-mediated uptake of the therapeutic enzyme from the blood into target tissues. Another approach under development is lipid nanoparticle mRNA–mediated gene therapy to express human GDE in the liver. However, this mRNA treatment will be effective only in the liver and requires repeated drug administration (29).

AAV9 can reliably transduce the major affected tissues in GSD III, thus providing an optimal treatment approach with established safety and efficacy profiles from numerous preclinical studies in multiple animal models and clinical trials (11, 12, 30–32). In this study, we designed an innovative LSP-CB dual promoter consisting of a tandem LSP and a ubiquitous promoter. We hypothesized that this dual promoter would retain the ability of the LSP to induce immunotolerance to pullulanase and enable therapeutic pullulanase expression in all affected tissues from the ubiquitous CB promoter. Intravenous injection of the AAV9-Dual-Pull vector at a dose of 5 × 10^12^ vg/kg in adult GSD IIIa mice effectively reduced pullulanase-related CTL responses and achieved efficacious pullulanase expression and glycogen correction in all major affected organs, including liver, heart, and skeletal muscles, after 10 weeks of treatment. However, this treatment did not correct glycogen accumulation in the smooth muscle and CNS (Supplemental Figure 1). Quantitation of vg at 2 weeks after AAV injection showed that the copy number of AAV-Dual-Pull (vg/diploid genome) was extremely high in the liver (325.6 ± 15.92 vg/diploid genome, Figure 2A), moderate in the heart (3.39 ± 0.46 vg/diploid genome, Figure 2A) and quadriceps (2.13 ± 0.21 vg/diploid genome, Figure 2A), and relatively low in the bladder (0.74 ± 0.22 vg/diploid genome, data not shown), small intestine (1.55 ± 0.034 vg/diploid genome, data not shown), and brain (0.60 ± 0.11 vg/diploid genome, data not shown). Further analysis with these tissues by Western blot showed that pullulanase protein bands were strong in the heart, very weak in the quadriceps, and undetectable in the bladder, small intestine, and brain (data not shown). The failure of AAV-Dual-Pull to express pullulanase in the smooth muscles and CNS might be a result of weak promoter or rapid clearance of the exogenous protein in the cells.

It is worth noting that AAV-LSP-Pull showed a higher efficiency than AAV-Dual-Pull in preventing transgene-induced CTL responses (Figure 7) and in correcting liver glycogen accumulation (Figure 5) in adult GSD IIIa mice, suggesting that AAV-LSP-Pull is a better choice for gene therapy in patients with GSD IIIb who have only liver involvement. Compared with the AAV-LSP-Pull treatment, we did see weak CTL responses in the AAV-Dual-Pull–treated mice (Figure 7). Therefore, further optimization of the dual promoter and a longer-term experiment are needed to determine the durability of the dual promoter with regard to the prevention of T cell responses to pullulanase and to achieve persistent therapeutic enzyme expression and glycogen correction in GSD IIIa mice. Colella et al. have previously reported the use of a liver-muscle tandem promoter to provide combined hepatic and extrahepatic muscle-specific transgene expression that prevented anti-transgene immunity in mice with Pompe disease (33). Systemic administration of an AAV9 or AAV8 vector containing the liver-muscle tandem promoter in GAA-KO mice resulted in persistent therapeutic efficacy in muscle (33). This approach could also be applied for the treatment of GSD IIIa, as liver and muscle are the major affected tissues.

Previously, we have demonstrated that coinjection of the AAV8-LSP-hGAA and AAV8-CB-hGAA vectors in adult GAA-KO mice can induce immune tolerance to hGAA by the LSP promoter (22). However, this approach was not efficacious in our GSD IIIa mice cotreated with the AAV9-LSP-Pull and AAV9-CB-Pull vectors. The reason for this discrepancy is likely that hGAA is a secreted protein, while pullulanase is a nonsecreted protein that would require administration of a much higher dose of the LSP-containing vector to induce immune tolerance. Perrin et al. demonstrated that, compared with an AAV8 vector expressing a secreted OVA, a 10-fold increase in vector dose was required for an AAV8 vector expressing cytoplasmic bound OVA to induce a comparable frequency of OVA-specific Tregs in mice (34).

In conclusion, we demonstrated that both the LSP and dual promoter significantly reduced pullulanase-specific CTL responses in adult GSD IIIa mice. Gene therapy with the AAV9-LSP-Pull vector for 10 weeks effectively cleared glycogen accumulation in the liver but not in other tissues, while the AAV9-Dual-Pull vector significantly reduced glycogen levels in the liver, heart, and skeletal muscles. Future studies are needed to determine the long-term durability of the dual promoter–mediated expression of pullulanase in adult GSD IIIa mice and in large animal models.

## Methods

### AAV vector constructs and AAV viral packaging.

To generate the pAAV-LSP-CB-Pull vector plasmid, the LSP promoter that contains a thyroid hormone-binding globulin promoter sequence, 2 copies of an α-1-microglobulin/bikunin enhancer sequence, and a leader sequence (35) was amplified from the pAAV-LSP-Pull vector using primers, *Xba*I-LSP-F and *Afl*II-LSP-R (Supplemental Table 1), and the CB promoter was amplified from the pAAV-CB-Pull vector (14) using primers *Afl*II-CB-F and *Kpn*I-CB-R (Supplemental Table 1). The amplified *Xba*I-LSP-*Afl*II and *Afl*II-CB-*Kpn*I fragments were ligated through the *Afl*II site and amplified again using primers *Xba*I-LSP-F and *Kpn*I-CB-R (Supplemental Table 1). The amplified *Xba*I-LSP-CB-*Kpn*I fragment was cloned into the pAAV-LSP-Pull vector at the *Xba*I and *Kpn*I sites to replace the LSP promoter. The pAAV-LSP-Pull, pAAV-CB-Pull, and pAAV-LSP-CB-Pull vectors were packaged as AAV9 (packaging plasmid was provided by James M. Wilson, University of Pennsylvania, Philadelphia, Pennsylvania, USA) in HEK293T cells (original cells from the Frank Graham laboratory, McMaster University, Hamilton, Ontario, Canada) using the calcium phosphate-triple transfection method and purified using the iodixanol gradient ultracentrifugation method (14, 36). The titer of the viral stock was determined by Southern blot using purified viral DNA and a biotin-labeled probe generated with Prime-A-Gene labeling kit (Promega). The viral vector stock was handled according to Biohazard Safety Level 2 guidelines published by the NIH.

### Animals and viral vector administration.

The GSD IIIa (*Agl*-KO) mouse model was generated by deleting exons 6–10 in the *Agl* gene as described previously (14). Ten-week-old GSD IIIa mice were injected with AAV9-LSP-Pull, AAV9-CB-Pull, AAV9-LSP-Pull + AAV9-CB-Pull coadministration, or AAV9-LSP-CB-Pull at the same dose, 5.0 × 10^12^ vg/kg (Figure 1A). After 2 or 10 weeks of treatment, the mice were sacrificed to collect tissues and blood following overnight fasting. The mice were examined by treadmill test at 2, 3, 4, and 5 months of age. Sex- and age-matched UT GSD IIIa mice and WT mice were used as controls. Fresh tissue specimens were either immediately frozen on dry ice and stored at –80°C until used for biochemical analyses or fixed immediately for histology.

### AAV vector biodistribution.

AAV vg were quantified by real-time PCR. Genomic DNA was extracted from frozen tissues using the Wizard Genomic DNA Purification kit (Promega, Madison, WI). PCR was performed using SYBR Green (Roche) and the gene-specific primer pairs for pullulanase and mouse β-actin (Supplemental Table 1). The pAAV-CB-Pull plasmid DNA was used to generate a standard curve for calculating viral vector copy numbers.

### Pullulanase activity assay.

Pullulanase activity was measured using the pullulanase activity assay kit (Megazyme) following the manufacturer’s protocols. Briefly, tissues were homogenized using a tissue homogenizer (Caframo LTD) on ice in PBS containing a protease/phosphatase inhibitor cocktail (Cell Signaling Technology). After centrifugation at 18,000*g* at 4°C for 15 minutes, the lysates were incubated with the substrate (6-O-Benzylidene-4-nitrophenyl-63-α-D-maltotriosyl-maltotriose), thermostable α-glucosidase, and thermostable β-glucosidase at 40°C. After 10 minutes, the reaction was stopped by adding the stop buffer (2% [w/v] Tris buffer, pH 9.0). The absorbance was read at 405 nm using a victor X multilabel plate reader (PerkinElmer). Kit-provided pullulanase enzyme was used as a standard to determine the enzyme activity. Protein concentrations in tissue lysates were determined by BCA assay and used to normalize the data.

### Glycogen content assay.

The same lysates from pullulanase activity assay were diluted 1:5 in distilled water and boiled for 3 minutes to inactivate endogenous enzymes. The diluted samples were incubated with 0.175 U/mL (final concentration in the reaction) of Amyloglucosidase (Sigma-Aldrich Co.) for 90 minutes at 37°C. The reaction mixtures were then boiled again for 3 minutes to stop the reaction. 30 μL of the mixtures was incubated with 1 mL Pointe Scientific Glucose (Hexokinase) Liquid Reagents (Fisher) for at least 10 minutes at room temperature. The absorbance at 340 nm was read using a UV-VIS Spectrophotometer (Shimadzu UV-1700 PharmaSpec).

### Histology.

Fresh tissues were fixed immediately in 10% neutral-buffered formalin for 48 hours. After primary immersion fixation, the samples were post-fixed with 1% periodic α in 10% neutral-buffered formalin for 48 hours at 4°C. The samples were then washed with PBS, dehydrated with ascending grades of alcohol, cleared with xylene, and infiltrated with paraffin. For PAS staining, sections of paraffin-embedded tissues were processed and stained using Schiff reagent as described previously (14). Briefly, the slides were oxidized with freshly made 0.5% periodic α for 5 minutes and rinsed with distilled water for 1 minutes. The slides were then stained with Schiff reagent for 15 minutes and washed with tap water for 10 minutes. The slides were counterstained with hematoxylin and rinsed with tap water, incubated with bluing reagent for 1 minutes, dehydrated, and mounted. For trichrome staining, the paraffin-embedded liver sections were processed and stained using Masson’s trichrome staining kit (Sigma-Aldrich) following the manufacturer’s protocols. Images were taken on a BZ-X710 microscope (Keyence America).

### Plasma enzyme activity tests.

The activities of plasma ALT, AST, and CK were measured using the Liquid ALT (SGPT), Liquid AST (SGOT), and Liquid Creatine Kinase Reagent Set, respectively (Pointe Scientific Inc.). Whole blood was collected in a green blood collection tube (coated with lithium heparin), and plasma was separated by centrifugation at 2,000*g*, 4°C, for 10 minutes and diluted (1:5) with normal saline (0.9% [w/v] of NaCl). The diluted plasma was incubated with the working reagent (R1 and R2 mixture) at 37°C, and absorbance at 340 nm was recorded every minute for 5 minutes.

### Treadmill exhaustion test.

Mice were acclimated for 15 minutes in the chamber of the treadmill (LE8709, Panlab Harvard Apparatus) and warmed up by running at the lowest speed, 5 cm/s, and 25 degrees of slope for 3 minutes. Then, mice were allowed to run at 8 cm/s for 3 minutes. The speed was increased by 4 cm/s every 3 minutes until mice were exhausted or the maximal speed (32 cm/s) was reached.

### Immunohistochemistry.

Paraffin-embedded sections were deparaffinized and rehydrated. The slides were incubated in Tris-EDTA (pH9.0) buffer at 100°C for 20 minutes for antigen retrieval, and the slides were washed with cold tap water and TBS containing 0.025% Triton X-100 (TBST). The samples were incubated with 10% normal goat serum with 1% BSA in TBS for 2 hours at room temperature, and primary antibodies were diluted in 1%BSA/TBS at 4°C overnight. The following primary antibodies were used: recombinant anti-CD4 antibody (Abcam, ab183685) and recombinant anti-CD8α antibody (Abcam, ab209775). The next day, the slides were washed with TBST, incubated with 0.3% H_2_O_2_ in TBS for 15 minutes, and incubated with HRP-conjugated secondary antibody for 1 hour at room temperature. The samples were washed and developed with SignalStain DAB substrate Kit (Cell Signaling Technology). The slides were rinsed, counterstained with hematoxylin, dehydrated, cleared, and mounted. The images were taken on a BZ-X710 microscope (Keyence America).

### Splenocytes isolation.

Fresh spleens were immediately collected from GSD IIIa mice and submerged in RPMI 1640 media (Gibco, Thermo Fisher Scientific). The spleen was carefully minced with the end of a syringe plunger in a culture dish with RPMI 1640 media and then the supernatant mix was transferred into a 70 μm cell strainer (Corning Inc.) sitting on a 50 mL conical tube. The cells and dissociated tissue were gently passed through the strainer by pressing them in a circular motion with a new syringe plunger and washing the strainer with culture medium. The cells were centrifuged at 350*g* for 10 minutes, and the supernatant was carefully discarded. The cells were resuspended in the medium, and the cells were incubated with 1× BD Pharm Lyse lysing solution for 15 minutes to disrupt red blood cells. The cells were centrifuged at 350*g* for 10 minutes, and the supernatant was carefully discarded. The cells were washed and resuspended with RPMI 1640 media, and cell numbers were counted using Turk’s dye (Ricca Chemical Company) and Erythrosin B dye (Thermo Fisher Scientific) to exclude dead cells.

### Mouse IFN-γ ELISpot.

Mouse IFN-γ secretion in response to the stimulation of pullulanase peptides was determined using an ELISpot assay kit (R&D Systems) following the manufacturer’s protocols. Briefly, the final pullulanase peptide pool (Supplemental Methods) was diluted in the culture medium to a final concentration of 4 μg/mL. The diluted peptide pool was added to a 96-well plate (100 μL/well), and then 100 μL splenocytes (1 × 10^7^ cells/mL) was added to each well. Medium only and concanavalin A (Thermo Fisher Scientific) were used as negative and positive controls, respectively. Cells were incubated overnight at 37°C. The number of SFU in each well was counted using an automated ELISpot reader (ImmunoSpot Analyzer S6 Universal M2, CTL).

### Statistics.

Data were analyzed using Prism GraphPad. Data normality was determined by Shapiro-Wilk test. For data with normal distribution, statistical significance was determined by ordinary 1-way ANOVA with post hoc test (Tukey’s); for data with nonnormal distribution, 1-way nonparametric ANOVA (Kruskal-Wallis) with post hoc test (Dunn’s) was used. Data are presented as mean ± SD. *P* values of less than 0.05 were considered statistically significant.

### Study approval.

The present studies in animals were reviewed and approved by the Duke University Institutional Animal Care and Use Committee review board, and animal care and experiments were conducted following approved guidelines.

## Author contributions

BS developed the concept of the dual promoter. BS and PSK designed the study. JAL performed experiments and interpreted the data. JAL, BS, and PSK wrote the manuscript.

## Supplementary Material

Supplemental data

## Version 1 10/20/2022

In-Press Preview

## Version 2 12/08/2022

Electronic publication
