# Supplementary material for: Suppression of pullulanase-induced cytotoxic T cell response with a dual promoter in GSD IIIa mice
Source: JCI Insight. 2022 Dec 8;7(23):e152970. doi: 10.1172/jci.insight.152970 (PMC9746900; doi:10.1172/jci.insight.152970)
Supplement: Supplemental data [file jciinsight-7-152970-s099.pdf]

SUPPLEMENTAL MATERIALS

Supplemental methods

Generation of Pullulanase peptide library and screening of peptide pools by IFN-γ ELISpot

A Pullulanase peptide library was designed to consist of 15-mer peptides, each overlapping by 10 amino acids with adjacent peptides. A total of 142 peptides that cover the entire peptide sequence of Pullulanase were synthesized at GenScript (Piscataway, NJ). Each peptide was dissolved in 100% DMSO to a final concentration of 10 mg/mL and a total of 24 Pullulanase peptide pools were generated using a two-dimensional array (12 ×12) with individual peptides, as shown in **Supplemental Figure 3** (Wang et al, Methods Mol Biol, 2012. https://www.ncbi.nlm.nih.gov/pubmed/21956501). Each peptide pool was prepared by combining an equal quantity of each indicated peptide stock along an axis to a final peptide concentration of 2 mg/mL. The 24 peptide pools underwent two rounds of screening by mouse IFN-γ ELISpot using splenocytes isolated from the CB-treated mice (two-week treatment), to identify immunogenic peptides. A final Pullulanase peptide pool consisting of eight of the most immunogenic peptides was selected to compare Pullulanase-specific T cell responses between the AAV treatment groups by ELISpot.

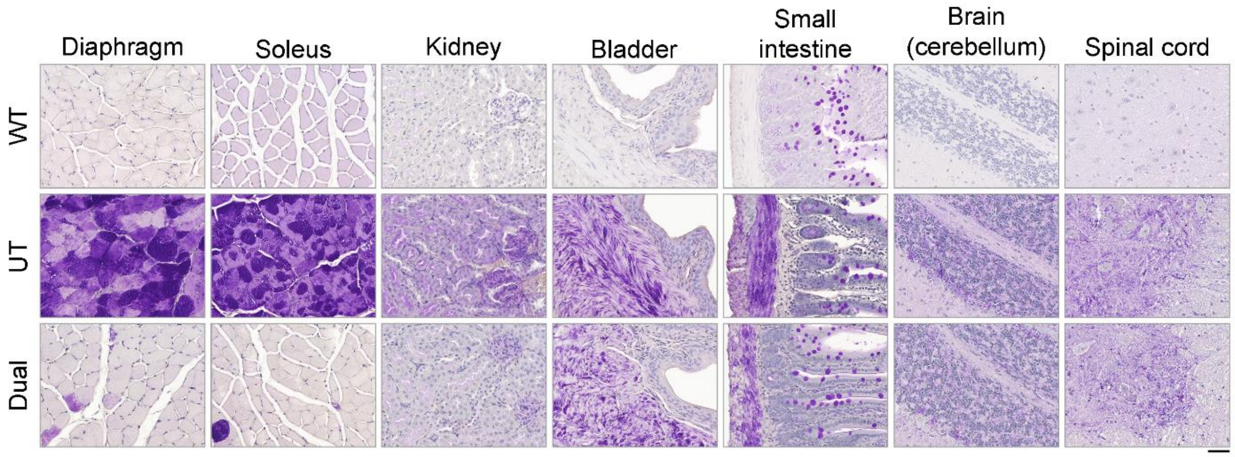

Supplemental Figure 1. Ten-week treatment with AAV-Dual-Pull cleared glycogen accumulation in other skeletal muscles and the kidney but had little effect on the smooth muscle and CNS. PAS-stained tissue sections revealed that the AAV-Dual-Pull treatment cleared accumulated glycogen from the diaphragm, soleus muscle, and kidney, but not in the bladder, small intestine, brain, and spinal cord. The images represent at least three mice in each group. Bar=50 µm.

	1	2	3	4	5	6	7	8	9	10	11	12
13	1	2	3	4	5	6	7	8	9	10	11	12
14	13	14	15	16	17	18	19	20	21	22	23	24
15	25	26	27	28	29	30	31	32	33	34	35	36
16	37	38	39	40	41	42	43	44	45	46	47	48
17	49	50	51	52	53	54	55	56	57	58	59	60
18	61	62	63	64	65	66	67	68	69	70	71	72
19	73	74	75	76	77	78	79	80	81	82	83	84
20	85	86	87	88	89	90	91	92	93	94	95	96
21	97	98	99	100	101	102	103	104	105	106	107	108
22	109	110	111	112	113	114	115	116	117	118	119	120
23	121	122	123	124	125	126	127	128	129	130	131	132
24	133	134	135	136	137	138	139	140	141	142		

Supplemental Figure 2. Illustration of a two-dimensional array for generation of Pullulanase peptide pools. A total of 142 peptides that covers the entire Pullulanase sequence were used to generate 24 peptide pools, each containing the indicated peptides in the corresponding columns or rows. A two-round screening by mouse IFN-γ ELISpot was conducted using the splenocytes isolated from the CB-treated mice (positive controls), to identify the most immunogenic peptides. A final Pullulanase peptide pool consisting of eight of the most immunogenic peptides was used to compare Pullulanase-specific T cell responses between the AAV treatment groups.

Supplemental Table 1. PCR primer pairs used in this study

Target		Name	Sequences
LSP	Forward	XbaI-LSP-F	5'-AGTTCTAGAGCGGCCGCCAG-3'
	Reverse	AflII-LSP-R	5'-CCCCTTAAGCCATTTTTATAGCATGTCCTGTATTGCAAAACTA-3'
CB	Forward	AflII-CB-F	5'-CCCCTTAAGGTTCCGCGTTACATAACTTACGGTAAAT-3'
	Reverse	KpnI-CB-R	5'-GTCGACGGTACCGCGCAG-3'
Pullulanase	Forward	Pull-F	5'-GCCACTGGATGCCTACAACT-3'
	Reverse	Pull-R	5'-CGTGCTGGTGCAGTGTATTG-3'
β-Actin	Forward	Actin-F	5'-AGAGGGAAATCGTGCGTGAC-3'
	Reverse	Actin-R	5'-CAATAGTGATGACCTGGCCGT-3'
